# Coupling/Uncoupling Reversibility in Isolated Mitochondria from *Saccharomyces cerevisiae*

**DOI:** 10.3390/life11121307

**Published:** 2021-11-27

**Authors:** Lilia Morales-García, Carolina Ricardez-García, Paulina Castañeda-Tamez, Natalia Chiquete-Félix, Salvador Uribe-Carvajal

**Affiliations:** 1 Department of Genetics and Molecular Biology, Instituto de Fisiología Celular, UNAM, Mexico City 04510, Mexico; lmoga15@gmail.com (L.M.-G.); cricardez@ifc.unam.mx (C.R.-G.); paulinact08@gmail.com (P.C.-T.); nchiquete@ifc.unam.mx (N.C.-F.); 2 Department of Biochemistry, Medicine School, UNAM, Mexico City 04510, Mexico

**Keywords:** physiological uncoupling, yeast mitochondria, mitochondrial permeability transition reversibility, *_Sc_*MUC, Ca^2+^

## Abstract

The yeast *Saccharomyces cerevisiae* uses fermentation as the preferred pathway to obtain ATP and requires the respiratory chain to re-oxidize the NADH needed for activity of Glyceraldehyde-3-phosphate. This process is favored by uncoupling of oxidative phosphorylation (OxPhos), which is at least partially controlled by the mitochondrial unspecific pore (*_Sc_*MUC). When mitochondrial ATP synthesis is needed as in the diauxic phase or during mating, a large rise in Ca^2+^ concentration ([Ca^2+^]) closes *_Sc_*MUC, coupling OxPhos. In addition, *_Sc_*MUC opening/closing is mediated by the ATP/ADP ratio, which indicates cellular energy needs. Here, opening and closing of *_Sc_*MUC was evaluated in isolated mitochondria from *S. cerevisiae* at different incubation times and in the presence of different ATP/ADP ratios or varying [Ca^2+^]. Measurements of the rate of O_2_ consumption, mitochondrial swelling, transmembrane potential and ROS generation were conducted. It was observed that *_Sc_*MUC opening was reversible, a high ATP/ADP ratio promoted opening and [Ca^2+^] closed *_Sc_*MUC even after several minutes of incubation in the open state. In the absence of ATP synthesis, closure of *_Sc_*MUC resulted in an increase in ROS.

## 1. Introduction

Fermentative metabolism in *Saccharomyces cerevisiae* requires an active mitochondrial respiratory chain to re-oxidize the NADH produced by glyceraldehyde-3-phosphate dehydrogenase (GAPDH) [1]. NAD^+^ is needed in the glycolytic pathway by glyceraldehyde-3-Phosphate dehydrogenase (GAPDH), which may bind to mitochondrial porin and exchange produced NADH for required NAD^+^ [2]. A basal high rate of oxygen consumption is possible in *S. cerevisiae* mitochondria due to the substitution of Complex I by three alternative NADH dehydrogenases (NDH2), which do not pump protons and thus decrease the ATP/O [3]. In addition, as Complex-I contains the two most important sites for ROS production in the respiratory chain [4] this substitution probably helps to avoid ROS overproduction in *S. cerevisiae*. In addition, the *S. cerevisiae* mitochondrial unspecific channel (*_Sc_*MUC) may open, depleting the transmembrane potential (ΔΨ), uncoupling oxidative phosphorylation and increasing the rate of oxygen consumption, thus decreasing ROS production further [5,6,7].

When fermentative substrates are exhausted and O_2_ is available, *S. cerevisiae* cells shift their metabolism to become aerobic using accumulated ethanol as respiratory substrate to begin the diauxic phase of growth [8,9]. Physiologically important effectors that alternatively open and close *_Sc_*MUC are adenine nucleotides, where ATP opens *_Sc_*MUC [7] while hydrolysis products ADP and Pi close it, i.e., a high energy charge opens *_Sc_*MUC while a low energy charge closes it. The second effector of physiological interest is Ca^2+^, which closes *_Sc_*MUC at high concentrations [10]. In resting yeast, cytoplasmic [Ca^2+^] is near 0.1 μM, while in most extracellular media [Ca^2+^] is 1 to 2 mM [11]. Upon stimulation, specialized channels allow large amounts of Ca^2+^ to enter the cell, closing *_Sc_*MUC and enhancing energy production [12]. Thus, Ca^2+^ is an ideal second messenger [12,13]. Activation by Ca^2+^ influx is observed during mating: when an a-type haploid cell is exposed to α-pheromone, this pheromone binds to a specific receptor [14]. When mating, cytoplasmic [Ca^2+^] may remain high for up to 20 min signaling the need large morphological changes in the cell due to large modifications in the cellular cytoskeleton [14]. Eventually, an a-cell and an α-cell undergo fusion to generate a diploid [15]. As these processes require a large amount of energy, Ca^2+^ is also needed to close *_Sc_*MUC, promote OxPhos coupling and increase ATP availability. When the mating process is over, Ca^2+^ is expelled and a basal metabolism is reinstated [13,16].

Here, we tested in isolated mitochondria the response to either the ratio ATP/ADP + Pi or to [Ca^2+^] variations. The role of each effector to control alternative opening and closing was followed using measurements of the rate of O_2_ consumption, mitochondrial transmembrane potential (ΔΨ), mitochondrial swelling and ROS production. The open/close state of *_Sc_*MUC was dependent on the addition of different effectors. Opening and closing were reversible. In addition it was observed that opening was gradual as effector mixtures elicited partial effects. These effects suggest that in fermenting yeast, mitochondria are uncoupled, becoming coupled when a large amount of ATP is required.

## 2. Materials and Methods

Reagents. All chemicals were analytic grade. Mannitol, MES hydrate, D-Galactose, TEA (triethanolamine), ADP, ATP, safranine-O, oligomycin were from Sigma Chem Co. Bovine serum albumin from Probulmin TM. Bacto-peptone and yeast extract from MCD LAB. H_3_PO_4_, KCl and MgCl_2_ were from J.T. Baker.

Yeast strain. Experiments were conducted using either a commercial strain of baker’s yeast *Saccharomyces cerevisiae* (“La Azteca, S. A.” Mexico City) [17] or a laboratory strain W303 (*MATα; ura3-1; trp1Δ 2; leu2-3,112; his3-11,15; ade2-1; can1-100*) [18]. Results were similar for both strains. Yeasts were kept in YPD (1% yeast extract, 2% bacto-peptone, 2% glucose and 2% bacto-agar) plate cultures. Cells were grown as follows: pre-cultures were prepared immersing a loophole of yeast into 100 mL YPD and incubating for 24 h under continuous agitation in an orbital shaker (New Brunswick Scientific, NJ, USA) at 250 rpm in a constant-temperature room (30 °C). Then each 100 mL flask was used to inoculate 900 mL of YPGal (1% yeast extract, 2% bacto-peptone and 2% galactose). Incubation was continued for 48 h.

Isolation of yeast mitochondria. After incubation, yeast was centrifuged (5000× *g* for 5 min and washed twice) and resuspended in 0.6 M mannitol, 5 mM MES, 0.1% bovine serum albumin, pH 6.8 (TEA). The cell suspension was mixed with 0.5 mm diameter glass beads 50% (*v*/*v*) and disrupted in a Bead Beater, Biospec Products Inc, OK. Mitochondria were isolated from the homogenate by differential centrifugation as previously described [17]. The concentration of mitochondrial protein was determined by Biuret [19]. In all assays we used mitochondria at a final concentration of 0.5 mg prot/mL.

Oxygen consumption. Experiments were conducted using a Clark electrode (Oximeter model 782, Warner/Strathkelvin Instruments, North Lanarkshire, Scotland) in a water-jacketed chamber. Temperature was kept at 30 °C using a water bath (PolyScience 7 L, IL). Total volume 1.0 mL. The reaction mixture was 0.6 M mannitol, 5 mM MES (TEA), pH 6.8 plus 0.1 M KCl, 0.5 mM MgCl_2_ and 2 μL/mL ethanol. In all experiments, samples were preincubated for 5 min with oligomycin 4 μg/mg prot.

Transmembrane potential. ΔΨ was determined as described by Åkerman and Wikström [20], following the changes in absorbance of safranine-O at 511–533 nm using a double beam Aminco-Olis spectrophotometer (GA) in dual mode. The concentrations of ATP, ADP, Ca^2+^ and EGTA are indicated under each figure. At the end of each trace, the collapse of ΔΨ was induced adding 6 μM CCCP. In all experiments, samples were preincubated for 5 min with oligomycin 4 μg/mg prot.

Mitochondrial swelling. The decrease in absorbance of a mitochondrial suspension at 540 nm was followed as described in the literature [6]. We used a DW2000 Olis/Aminco spectrophotometer in split mode. In all experiments, samples were preincubated for 5 min with oligomycin 4 μg/mg prot.

Reactive oxygen species were measured in freshly mitochondria using the Amplex Red (Invitrogen, Molecular Probes, Carlsbad, CA, USA) as in [21]. Samples were incubated for 1 min, then 50 µg were placed into a 96-well micro plate with working solution 20 µL (10 µM Amplex red, 0.2 units/mL horseradish peroxidase and 0.2 units superoxide dismutase/mL in 250 mM sodium phosphate pH 7.4), final volume 100 µL. Fluorescence was measured after 30 mins in a POLARstar Omega detector (BGM LABTECH, Offenburg, Germany) set at 571 and 585 nm and results were interpolated against a calibration curve.

## 3. Results

During fermentation, *Saccharomyces cerevisiae* uncouples OxPhos to oxidize NADH optimizing the rate of glycolysis while in contrast, after glucose depletion a coupled OxPhos is needed and thus *_Sc_*MUC is closed [8]. Among *_Sc_*MUC effectors, the molecules that vary in concentration in different metabolic states such as adenine nucleotides and Pi seem important. Indeed, ATP opens *_Sc_*MUC uncoupling OxPhos, while ADP closes it, coupling OxPhos [22]. Another effector is [Ca^2+^], which in eukaryotes increases several orders of magnitude when plasma membrane Ca^2+^ channels open [12,13,14].

**Figure 1:**
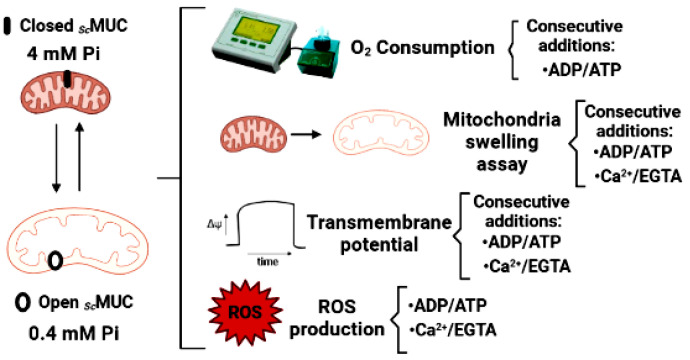
Experimental procedures followed to determine the possible physiological role of *_Sc_*MUC in isolated *S. cerevisiae* mitochondria. Initially, *_Sc_*MUC was open in the presence of 0.4 mM Pi or closed in the presence of 4.0 mM Pi. Then different concentrations of the antagonizing effectors ADP, which closes *_Sc_*MUC or ATP, that opens it, were added, Otherwise, opening or closing was promoted by adding Ca^2+^ to close *_Sc_*MUC or EGTA to chelate the cation and open the channel. Under these conditions, experiments were performed to measure the rate of oxygen consumption, mitochondrial swelling, trasmembrane potential or ROS production.

To analyze the possible physiological role of *_Sc_*MUC, mitochondria were isolated and used to measure Oxygen consumption, mitochondrial swelling, the transmembrane potential (ΔΨ) and reactive oxygen species (ROS) production under conditions where *_Sc_*MUC was open or closed (Figure 1).

### 3.1. Effects of ADP/ATP on _Sc_MUC

**Figure 2:**
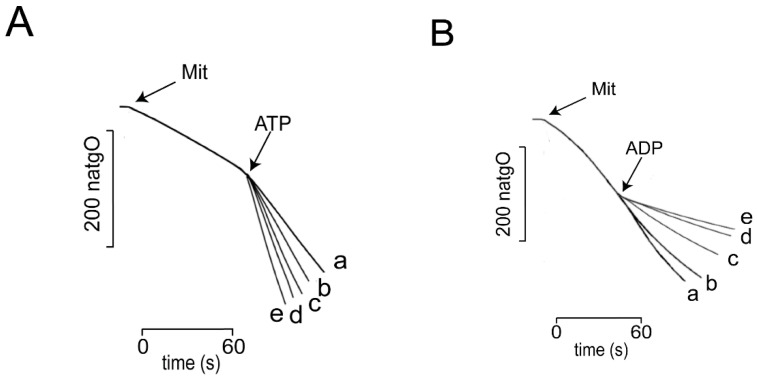
Rate of O_2_ consumption by isolated yeast mitochondria in the presence of different concentrations of phosphate (Pi), ATP and ADP. Reaction mixture: 0.6 M mannitol, 5 mM MES, pH 6.8, 0.1 mM KCl, 0.5 mM MgCl_2_, 2 μL/mL ethanol. In order to inhibit ATP/ADP interconversion, mitochondria (0.5 mg prot./mL) were incubated for 5 min in the presence of 4 μg oligomycin/mg prot. (**A**) Mitochondria were coupled in the presence of 2 mM Pi. Then, ATP was added at a final mM concentration of: (a) 0.5; (b) 1.0; (c) 1.5; (d) 2.0 and (e) 4.0. (**B**) To promote uncoupling, the experiment was conducted in the presence of 0.1 mM Pi. Then different concentrations of ADP were added to a final mM concentration of (a) 0.5; (b) 1.0; (c) 1.5; (d) 2.0 and (e) 4.0. Experiments were conducted in a Strathkelvin oxymeter equipped with a Clark electrode. T = 30 °C. Representative experiment, *n* = 3.

Oximetry. The rate of O_2_ consumption was measured in the presence of different, fixed Pi concentrations. Then the reversibility of *_Sc_*MUC opening in response to the addition of ATP and/or ADP was evaluated. As described in the literature [5,6,7], at 2.0 mM Pi we observed a slow rate of oxygen consumption, indicating that OxPhos was coupled (Figure 2A, trace a). Under these conditions, the addition of different concentrations of ATP proportionally increased the rate of oxygen consumption, suggesting that different concentrations of ATP gradually uncoupled OxPhos (Figure 2A, traces b to e). In contrast, as reported by others [5,6,7], at 0.1 mM Pi the rate of oxygen consumption was high, indicating that OxPhos was uncoupled due to opening of *_Sc_*MUC (Figure 2B trace a). Then, the rate of oxygen consumption gradually decreased upon addition of increasing concentrations of ADP, suggesting different ADP concentrations promoted closure of *_Sc_*MUC, promoting coupling (Figure 2B traces b to e). Oxygen consumption results confirmed results by others that increasing concentrations of Pi, and ADP close, while ATP opens *_Sc_*MUC [5,6,7].

**Figure 3:**
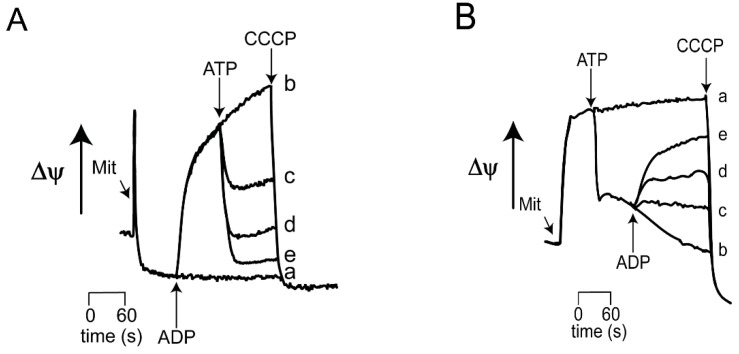
Effect of the sequential addition of ATP and ADP on the mitochondrial transmembrane potential (ΔΨ). (**A**) Reaction mixture as in Figure 2A, except 15 μM safranine-O was added. At the arrow, 1 mM ADP, except in trace a, where no ADP was added. Then 1.5 min later ATP was added as follows: traces a and b, 0; trace c, 0.5 mM; trace d, 1 mM; trace e, 1.5 mM. (**B**) Reaction mixture as in Figure 2B except 15 μM safranine-O. At the arrow, 1 mM ATP except in traces a and b, where no ATP was added. Then 1.5 min of incubation ADP was added as follows: Traces a and b, 0; trace c 0.5 mM; trace d, 1.0 mM; trace e, 1.5 mM. To deplete ΔΨ, 6 μM FCCP was added at the end of each trace. Absorbance measurements were conducted at 511–533 nm in a Olis/Aminco spectrophotometer in dual mode.

Transmembrane potential. The reversibility of *_Sc_*MUC opening and closing by ATP and ADP, respectively was evaluated measuring the ΔΨ (Figure 3). In these experiments, one adenine nucleotide was added first and the second was added 1.5 min later. At 0.1 mM Pi, ΔΨ was almost depleted (Figure 3A trace a) and addition of 2 mM ADP led to ΔΨ recovery (Figure 3A trace b). Then, the addition of increasing ATP led to a second fall in ΔΨ (Figure 3A, traces c, d and e). The opposite experiment was also performed: when in the presence of 2 mM Pi, ΔΨ was high (Figure 3B) and addition of ATP decreased ΔΨ (Figure 3B, traces b to e). Then, a later addition of different ADP concentrations resulted in increasing recovery of ΔΨ, suggesting that *_Sc_*MUC closed gradually (Figure 3B traces c, d and e). The reversibility in ΔΨ rise/decrease suggests that the alternative addition of ADP and ATP led to alternative, gradual opening/closing of *_Sc_*MUC.

**Figure 4:**
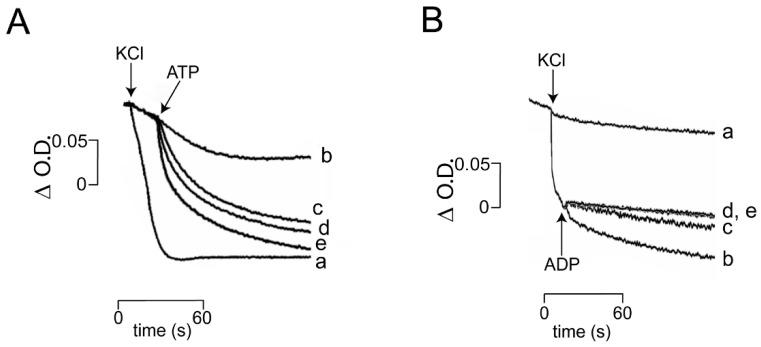
Effect of the sequential addition of ATP and ADP on mitochondrial swelling. (**A**): Reaction mixture, as in Figure 1, except 0.3 M mannitol. Where indicated 0.1 M KCl. (**A**) 0.1 mM Pi plus 1 mM ADP (ADP was not added in trace b). Where indicated, ATP was added as follows: (a) and (b) 0; (c) 1 mM; (d) 1.5 mM; (e) 2 mM. (**B**) 2 mM Pi plus 1 mM ATP ATP was not added in trace (a). Where indicated, ADP was added as follows: traces a and b, 0; trace c, 0.5 mM; trace d, 1 mM; trace (e) 2 mM.

Mitochondrial swelling. Another parameter commonly used to follow the open/close state of *_Sc_*MUC is mitochondrial swelling [6,23]. At low Pi plus ADP, *_Sc_*MUC was closed, so the rate of K^+^-mediated swelling was slow (Figure 4A). Under these conditions, addition of different concentrations of ATP promoted increasing rates in swelling. These data suggested that ATP reverted the ADP-mediated closing of *_Sc_*MUC. The opposite experiment, where mitochondria were incubated in the presence of 2 mM Pi plus 2 mM ATP, a rapid rate of K^+^-mediated swelling was observed which indicated *_Sc_*MUC was open (Figure 4B, trace b). Then, addition of different concentrations of ADP (Figure 4B, traces c, d, e) inhibited the rate of swelling (Figure 4B traces c, d, and e) suggesting that ADP closed *_Sc_*MUC. Therefore, the effect of Pi, ADP or ATP at different times led to changes of swelling rates suggesting that *_Sc_*MUC open and close states were reversible [24,25,26].

**Figure 5:**
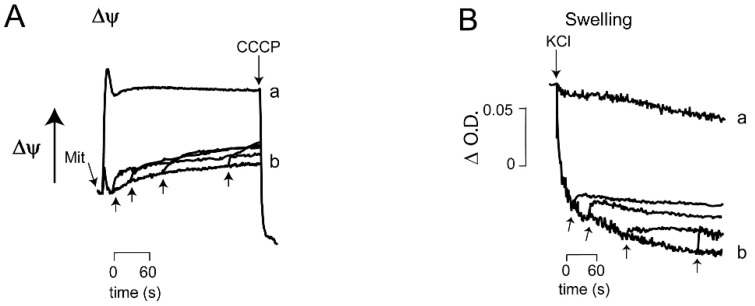
Partial reversion by ADP added at different times of the ATP-mediated opening of *_Sc_*MUC. Experimental conditions were as in Figure 3B for ΔΨ and Figure 4B for swelling. ATP was 0 (traces a) or 2 mM (all other traces). Upward arrows indicate addition of 1 mM ADP at different incubation times: 30 s, 1, 2 and 4 min. (**A**) Reaction mixture as in Figure 3B, except 1 mM ATP. (**B**): Reaction mixture, as in Figure 3B. Where indicated 0.1 M KCl. Representative experiment. *n* = 5.

Effects of incubation time on the reversibility ATP-mediated opening of _Sc_MUC. Preservation of *_Sc_*MUC opening reversibility after different incubation times is critical for survival of the cell. Reversibility of *_Sc_*MUC opening was explored measuring both ΔΨ (Figure 5A) and the rate of swelling (Figure 5B). Yeast mitochondria were incubated for different times, from 30 s to 4 min under open *_Sc_*MUC conditions, and then ADP was added (Figure 5). In all cases, ADP promoted partial recovery of ΔΨ (Figure 5A) and slight reversal of swelling (Figure 5B). Thus the ATP-mediated opening of *_Sc_*MUC was responsive to ADP for at least 4 min. (Figure 5).

### 3.2. Ca^2+^/EGTA Alternating Effects on _Sc_MUC

**Figure 6:**
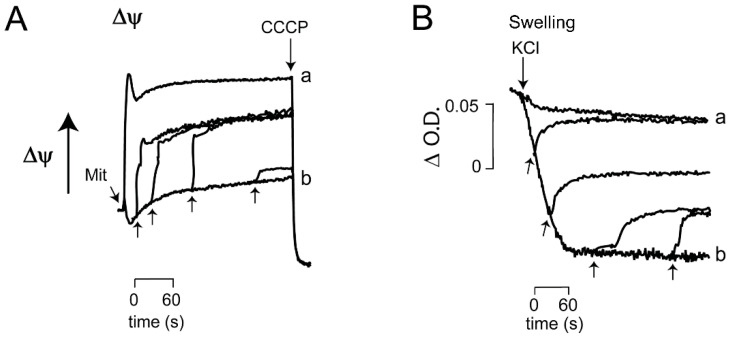
Effect of the addition of Ca^2+^ on mitochondrial transmembrane potential and mitochondrial swelling. (**A**) Reaction mixture as in Figure 4A. Ca^2+^ 600 μM (upward arrows) was added at: 30 s, 1, 2 and 4 min. (**B**) Reaction mixture as in Figure 4B. Where indicated 0.1 M KCl. (upward arrows).

The above results indicate that the opening response of *_Sc_*MUC to the sequential additions of ATP and ADP is reversible even after several minutes of incubation. However, neither mitochondrial ΔΨ nor swelling returned to the values observed when *_Sc_*MUC was closed from the beginning. Thus, it was decided to test whether full reversal of *_Sc_*MUC opening could be observed upon depletion of a positive effector after different times of incubation. From previous data, it was reasoned that this could be achieved with Ca^2+^, an effector closing *_Sc_*MUC, and its full chelation with EGTA to open *_Sc_*MUC [10,27]. To test whether the EGTA-mediated opening of *_Sc_*MUC was reverted adding Ca^2+^ at different incubation times, we conducted measurements of ΔΨ (Figure 6A) and mitochondrial swelling (Figure 6B). Mitochondria were incubated in the presence of EGTA for 0.5, 1, 2 and 4 min and then Ca^2+^ was added. Up to 2 min, the addition of Ca^2+^ promoted recovery of a large proportion of ΔΨ while a partial effect was obtained at 4 min. (Figure 6A) Mitochondrial swelling was reverted by Ca^2+^, indicating *_Sc_*MUC was closed (Figure 6B). The above data suggest that in isolated mitochondria from *S. cerevisiae*, Ca^2+^ chelation by EGTA evoked the reversible opening of *_Sc_*MUC. In contrast to the partial effects of ADP (Figure 5), Ca^2+^ addition resulted in better recovery of ΔΨ (Figure 6A) and also at variance with ADP, actual reversal of swelling (Figure 6B).

### 3.3. Reactive Oxygen Species (ROS) Production under Conditions That Open or Close _Sc_MUC

**Figure 7:**
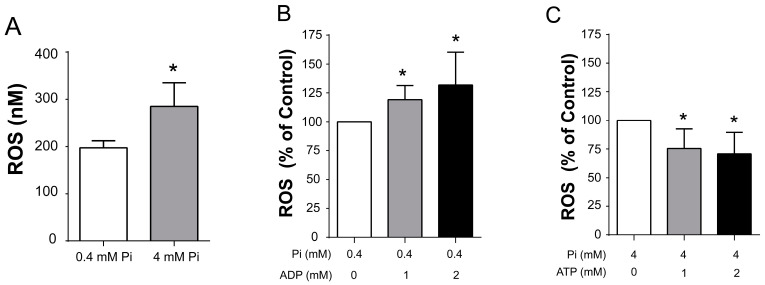

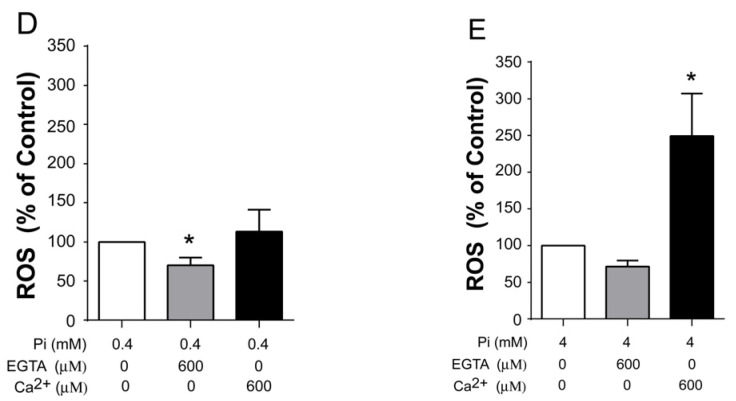
ROS production by *S. cerevisiae* mitochondria under different open/close states of *_Sc_*MUC. Production of peroxide was measured following resorufin fluorescence in a PolarStar Omega detector (571–585 nm); the results were interpolated against a calibration curve. (**A**) Total ROS in nM. Mitochondria incubated with 0.4 mM or 4.0 mM phosphate, data represent the mean ± standard deviation for three independent experiments (* *p* < 0.05, *t* test). From B to E, data are percentages of the control in (**A**,**B**) Mitochondria incubated with 0.4 mM phosphate with or without 1 or 2 mM ADP, (**C**) Mitochondria incubated with 4 mM phosphate with or without 1 or 2 mM ATP, (**D**) Mitochondria incubated with 0.4 mM phosphate with or without 600 µM EGTA or Ca^2+^ and (**E**) Mitochondria incubated with 4 mM phosphate with or without 600 µM EGTA or Ca^2+^. Data in (**B**–**E**) represent the mean ± standard deviation for three independent experiments (* indicates significant), Dunnett’s multiple comparisons test. (Note that the ROS percentage scale for figures (**B** and **C**) are different from (**D** and **E**). Raw data for this figure are in Supplementary Table S1.

Physiological uncoupling increases electron flow in the respiratory chain, preventing the over-production of ROS. It has been proposed that permeability transition pores work as unspecific proton sinks thus promoting physiological uncoupling and therefore decrease ROS production [5,6,7,25]. With this in mind, it was decided to determine whether a correlation exists between the opening/closing of *_Sc_*MUC reported above and the control of ROS production (Figure 7).

At 0.4 m Pi, ROS production was lower than at 4.0 mM Pi, suggesting as expected, that a closed *_Sc_*MUC enhanced ROS production (Figure 7A, Table S1). Then, to test other effectors used here, at 0.4 mM Pi, ADP was added at 1 or 2 mM, which closed *_Sc_*MUC (Figure 2,Figure 3,Figure 4,Figure 5), observing that ROS production increased (Figure 7B, Table S1). In contrast, in the presence of 4 mM Pi where *_Sc_*MUC was closed, addition of ATP, which opens *_Sc_*MUC (Figure 2,Figure 3,Figure 4,Figure 5), inhibited ROS production (Figure 7C, Table S1). Under the same low or high Pi concentrations, the effects of Ca^2+^ chelation or addition were tested. As expected from the results shown in Figure 6, at 0.4 mM Pi, EGTA decreased ROS production, while Ca^2+^ addition mildly increased ROS production (Figure 7D, Table S1). Then, at 4.0 mM Pi, EGTA inhibited ROS production, while Ca^2+^ addition resulted in more that twice as much produced ROS (Figure 7E, Table S1). Thus, when *_Sc_*MUC is closed ROS production is higher that when *_Sc_*MUC is open. These results are in agreement with the idea that an open *_Sc_*MUC is a physiological uncoupler of oxidative phosphorylation that inhibits production of ROS.

Together, our results add to the idea that the permeability transition protects mitochondria and thus the cell against stress. Effectors such as ATP and ADP probably control OxPhos in response with the energy charge, where high ATP signals for uncoupling. In addition, Ca^2+^ promotes a stronger response and prepares the cell in advance for high energy-requiring processes such as duplication of gene exchange with other cells [11,12,13] (Table 1).

## 4. Discussion

During fermentation, *S. cerevisiae* extracts all the energy it needs from glucose, while producing ethanol [28]. Under these conditions, ATP is high, so the F_1_F_0_ATP synthase is not required and thus it does not dissipate ΔΨ. A high ΔΨ would inhibit the rate of oxygen consumption by the respiratory chain (RC) [1], however RC does work at a high rate in order to oxidize NADH and produce the NAD^+^ needed for glyceraldehyde-3-phosphate dehydrogenase activity [29]. Therefore, during fermentation, mitochondrial OxPhos has to be uncoupled. Uncoupling is due to ATP itself, as it promotes opening of *_Sc_*MUC [5,6,7] (Figure 2,Figure 3,Figure 4). An open *_Sc_*MUC dissipates the mitochondrial ΔΨ, accelerating the rate of electron transfer in the respiratory chain [1]. In contrast, when fermentation substrates are depleted, ATP is hydrolyzed to ADP, which is a negative _Sc_MUC effector and thus promotes the recovery of a high ΔΨ [7,8]. Different reports [30,31,32] and data presented here (Figure 2,Figure 3,Figure 4), suggest that opening/closing of *_Sc_*MUC is not an all-or-nothing event, but instead partial opening states modulate the degree of uncoupling. Such modulation would be a mechanism to coordinate OxPhos and fermentation as needed [32]. Indeed, our results suggest that the degree of *_Sc_*MUC opening is gradual and depends on ATP/(ADP + Pi) concentrations (Figure 2,Figure 3,Figure 4, Figure 5). The physiological role of permeability transition may profit from the eventual elucidation of the structure of the pore, which seems to be approaching fast [33].

In addition to adenine nucleotides, Ca^2+^ is an important negative effector of *_Sc_*MUC. This contrasts with mammalian cells, where Ca^2+^ works in the opposite sense, i.e., in the mammalian mitochondrial permeability transition pore (mPTP) opening is triggered by Ca^2+^ [34,35]. In the resting state cytoplasmic Ca^2+^ is below 100 nM both in *S. cerevisiae*, and in mammalian cells, while external Ca^2+^ is in the mM range [11]. Several transport systems use this massive gradient to allow Ca^2+^ entry through specialized channels in the plasma membrane to the cytoplasm where it acts as a secondary messenger [36,37]. Again, at variance with most cells, where these transients last less than a minute, in *S. cerevisiae* cytoplasmic Ca^2+^ may remain high for as long as 20 min [12,13]. Thus, Ca^2+^ closes *_Sc_*MUC, enhancing OxPhos, while *_Sc_*MUC may remain open for several minutes and still be reversible. In contrast, in mammalians, mPTP opening is dangerous as it uncouples OxPhos and may eventually deplete ATP and trigger cell death through mitochondrial signaling [38].

In yeast, when an event requiring high amounts of energy occurs, Ca^2+^ enters the cell increasing ATP production. One such event may be the mating response, where massive morphologic changes occur that seem to consume a large amount of ATP [39]. Another event where Ca^2+^ enters the cell is phase S1 of the *S. cerevisiae* cell cycle when again it is likely that large amounts of ATP are needed in order to synthesize macromolecules [40]. Both mating and the S1 phase of the cell cycle are events where high mitochondrial ATP production is needed [16]. In these circumstances Ca^2+^ would enable OxPhos coupling by closing *_Sc_*MUC and increasing ΔΨ.

The mammalian mitochondrial permeability transition pore (mPTP) is widely considered as an equivalent entity to *_Sc_*MUC. Indeed, both mPTP and *_Sc_*MUC possess similar cutoff diameters [41] and react in similar fashion to some effectors such as adenine nucleotides [6,22] or to pharmacological agents such as bongkrekic acid or atractyloside [40]. However, the response to Ca^2+^ is the opposite as Ca^2+^ opens mPTP [42] while it closes *_Sc_*MUC [22,27]. In order to obtain coupled mammalian mitochondria, Mg^2+^ or Ca^2+^ chelators have to be added to the isolation medium, while this is not needed in yeast mitochondria [5,6,7,18]. Additionally, it must be considered that the structure of the channel is still under debate and some components that seem to participate in one channel are not present in the other. One such component may be PiC, which in yeast controls opening and confers sensitivity to Pi [24], but when manipulated in heart mice, it does not modify the properties of mPTP [43]. These results suggest that the still undefined components of mitochondrial permeability transition pores may be different for each species [44]. Furthermore, in mPTP, components of the Ca^2+^ uniporter machinery modify mPTP activity [45,46], while this is not possible in *S. cerevisiae* where there is no Ca^2+^ uniporter [47,48,49]. Thus, it is probably not safe to assume that mPTP and *_Sc_*MUC are identical entities.

In the mammalian heart, reversal of pore opening has also been reported [24,25,26,27], although opening of mPTP seems to react to different events than in *S. cerevisiae*. It is observed during cardiac stress, intermittent episodes of reperfusion allow the cell to empty Ca^2+^ while replenishing ATP pools. Thus, in mammals, mPTP seems important to prevent ROS overproduction through mitochondrial uncoupling, greatly improving survival probabilities [50]. This procedure is termed conditioning. Thus, even though both *_Sc_*MUc and mPTP constitute a mitochondrial uncoupling device, the role of *_Sc_*MUC opening is to oxidize NADH in a species that does not suffer from hypoxia [51].

Other yeast species also present some peculiarities that contribute to the idea that perhaps permeability transition pores are different. It is not clear whether *Yarrowia lipolytica* and perhaps *Endomyces magnusii* undergo mitochondrial permeability transition [52], as this event does not occur except under extreme circumstances, when a calcium ionophore plus massive amounts of calcium are added [53]. Another yeast, *Debaryomyces hansenii*, does undergo PT, however, its channel exhibits a unique sensitivity to Na^+^ and K^+^, which are negative effectors and such sensitivity is probably quite important as this yeast resides in marine environments and is used in salted cheese maturation [54].

In many species including *S. cerevisiae*, aging damages mtDNA, possibly through an excess in ROS that in turn increases activity of proteins that promote mtDNA recombination. Then mutations accumulate damaging mitochondria irreversibly in a “vicious cycle” [55,56].

Opening of *_Sc_*MUC increases the rate of O_2_ flow in the respiratory chain, preventing overproduction of ROS. This is not the only mechanism accelerating electron flow, as other proton sinks such as uncoupling proteins are differentially expressed upon exposure to stress [31]. A second mechanism promoting physiological uncoupling, where branched respiratory chains differentially express their alternative redox enzymes such as alternative oxidase (AOX) or NADH dehydrogenases type 2 (NDH2), in order to increase electron flux in the respiratory chain either at the beginning of the stationary phase or when the cell is subjected to stress [56,57].

The Index of Hydrogen Defficiency (IHF) is another exceedingly interesting mechanism mitochondria use to prevent damage by ROS. In aging *Endomyces magnusii* (grown for up to 168 h), unsaturated fatty acids are replaced by their saturated counterparts. So, as palmitic and oleic acid are less susceptible to damage by ROS than unsaturated fatty acids, mitochondrial membranes are protected. In addition, in this species, an alternative AOX is expressed from the beginning of the stationary phase, so physiological uncoupling is also used to protect mitochondria [58].

The “Co-localization for Redox Regulation (CoRR) Hypothesis” must also be considered when explaining how damaged mitochondria are negatively selected to allow cell survival [57]. When protection fails, mitochondria containing damaged mtDNA are negatively selected. This is possible due to the coexistence in the same compartment of mitochondrial DNA with its encoded proteins. These proteins are the most hydrophobic and difficult to move from the nucleus to the mitochondrial matrix. Additionally the CoRR hypothesis proposes that these mtDNA-encoded proteins are vital for the structure of OxPhos complexes I, III, IV and V. Therefore, deleterious mutations in mtDNA proteins would result in OxPhos malfunction and elimination of mitochondria [58].

Together, physiological uncoupling, IHF and mtDNA-encoded proteins (as proposed by the CoRR hypothesis), provide mitochondria with mechanisms to respond to exposure to stress, increased ROS and improve cell survival. Furthermore, regulation of *_Sc_*MUC by the energy charge and by Ca^2+^ signaling point to a role of *_Sc_*MUC in metabolic control, controlling fermentation, OxPhos and ROS production. In addition, a system to deplete O_2_ would confer yeast with an advantage over other organisms as few other cells thrive under hypoxic/anoxic conditions. Probably the equilibrium between ATP and ADP works to graduate *_Sc_*MUC responses, while Ca^2+^ signals an event where OxPhos has to be recruited.

## Figures and Tables

**Table 1:** Summary of Results.

Low or High Pi without Additions	Effector Additions
*_Sc_*MUC is OPEN at 0.4 mM Pi	and CLOSED by ADP 0.5 to 2 mM Ca^2+^ 100 to 600 μM
*_Sc_*MUC is CLOSED at 4.0 mM Pi	and OPENED by ATP 0.5 to 2.0 mM
At both Low and high Pi, alternating ADP/ATP close/open *_Sc_*MUC respectively	
At low Pi, Ca^2+^ addition/chelation close/open *_Sc_*MUC respectively	
Ca^2+^ is highly efficient to revert opening of *_Sc_*MUC, even after 4 min of incubation.	
DATA	
OPEN *_Sc_*MUC High Rate of Oxygen consumption Low ΔΨ Extensive mitochondrial swelling Low ROS generation	CLOSED *_Sc_*MUC Low rate of oxygen consumption High ΔΨ Low to nil mitochondrial swelling High ROS production

## Supplementary Materials

The following is available online at https://www.mdpi.com/article/10.3390/life11121307/s1: Table S1: Raw data used to generate Figure 7.
