# Supplementary material for: Coupling/Uncoupling Reversibility in Isolated Mitochondria from Saccharomyces cerevisiae
Source: Life (Basel). 2021 Nov 27;11(12):1307. doi: 10.3390/life11121307 (PMC8707985; doi:10.3390/life11121307)
Supplement: Supplementary file 1 [file life-11-01307-s001.zip › life-1420361-supplementary.pdf]

Table S1 Raw data used to generate Figure Seven.

Data for Figure 7 A. ROS Production by Yeast mitochondria, High and low Pi. Data are in nM.

Pi 0.4mM	Pi 4 mM
196.1765	249.3627
173.4314	255.8824
211.5441	267.0588
208.7754	240.8824
197.5	270.6373
220.5	361.0288
174.4	352.3218

Data for Figs 7B and 7C. ROS produced as % of the control at low or high Pi (Without ADP or ATP).

Pi 0.4mM			Pi 4mM		
	ADP			ATP	
	1mM	2 mM		1mM	2 mM
100	128.0812	126.2012	100	82.22921	88.81463
100	126.5823	128	100	65.55556	71.26437
100	120.7967	143.6951	100	63.06902	46.9163
100	134.4257	126.5823	100	77.92023	85.20554
100	120.7967	162.8889	100	67.45155	76.59844
100	134.4257	130.8864	100	53.72718	42.7514
100	100.6496	162.8889	100	78.65631	42.53025
100	96.28181	204.9024	100	117.769	63.10959
100	120.2072	105.7167	100	96.71491	60.75658
100	114.8211	111.9834	100	68.33903	62.80643
100	114.2579	114.2561	100	67.99471	87.86378
100		104.5198	100	84.38535	94.04134
100		120.0114	100	88.30636	60.99528
100		107.1956	100	52.10798	59.21803
			100	52.4525	95.29054
			100	85.65793	95.70666
			100	80.33186	

Data for Figs 7D and 7E. ROS produced as % of the control at low or high Pi (With EGTAS or Ca $^{2+}$).

Pi 0.4mM			Pi 4mM		
	EGTA 600 μM	Ca^{2+} 600 μM		EGTA 600 μM	Ca^{2+} 600 μM
100	64.54273	140	100	82.22921	297.5231
100	78.39175	69	100	65.55556	231.5517
100	56.5172	111	100	63.06902	190.6021
100	80.3002	132	100	77.92023	321.8254
100	70	115	100	67.45155	205.2346
